# Supplementary material for: Australian Mental Health Consumers’ Experiences of Service Engagement and Disengagement: A Descriptive Study
Source: Int J Environ Res Public Health. 2021 Oct 5;18(19):10464. doi: 10.3390/ijerph181910464 (PMC8508315; doi:10.3390/ijerph181910464)
Supplement: Supplementary file 1 [file ijerph-18-10464-s001.zip › ijerph-1297664-supplementary.pdf]

Supplementary File S1: Survey Questions

Q1. In which state/territory do you live?
Victoria
South Australia
New South Wales
Queensland
Tasmania
Western Australia
Northern Territory
Australian Capital Territory

Q2. Are you located in a
Capital City
Regional Centre
Remote Town

Q3. Are you
Male
Female
Other

Q4. What is your age range?
under 20 years
20 - 39 years
40 - 49 years
50-59 years
60-69 years
70-79 years
80 years or above

Q5. Are you of Aboriginal or Torres Strait Islander descent?
Yes
No
Prefer not to answer

Q6. What is your country of birth if not Australia?

Q7. What language do you mostly speak at home?
English
Other (please specify below)

Q8. Are you completing this survey as a:
Consumer (someone with a mental illness or experience of mental ill-health)?
Carer or Family Member

Q9. Select from the following options the one which best describes what services, health professional or supports you have mainly used in the past 5 years for your mental health
Public mental health services/hospitals/community teams
Private mental health services/hospitals
My GP
Only used a Private Psychiatrist
A Psychologist, counsellor/therapist
Veteran supports

Peer support (organised or unorganised)
Telehealth
Online or digital resources or Apps
Other (please specify)

Q10. Please explain the main reasons why you use this as your primary source of mental health support? Rate each
of the following reasons
Answer Choices a. Major Contributing Reason b.
Contributing Reason c. Not a Contributing Reason d. Not Applicable
I don't have to wait too long to see someone
The service meets my needs
They don't make me repeat my story too much
They listen to me
They include/collaborate with me
I feel I have some say or control in making decisions
They include my family/carer
They respect my privacy if I don't want to include my family
I trust them
I feel safe there
I don't feel judged / stigmatised by them
I can afford to pay for this service
Limited options/choice of service providers in my area
I have a consistent worker
They are organised and coordinate the support services I need
They seem to have a clear plan/goals
I am able to see a worker whose gender is of my choosing
Other (please specify)

Q11. If you used digital resources or Apps, which of the following influenced your decision to commence an online
course for mental health and wellbeing? (Select all that apply)
My health professional recommended that I do the course
My friends or family recommended that I do the course
It was convenient for me to access due to limited availability of other mental health services in my local area
It was convenient for me to access due to my limited availability to attend a face-to-face treatment
It was convenient for me to access outside of the normal consultation (business) hours
The cost of face-to-face services
I chose to remain anonymous and limit personal information shared
I wanted to control the level of contact I have with my service provider (e.g. no contact with doctor, only receive feedback via email)
I was on the wait list for other services
I previously used other services or treatments but was dissatisfied
I previously used or was still using other services but I wanted to try something new
I prefer to use digital services rather than face-to-face services
The reputation of the institutes providing the online course
The scientific evidence supporting the online course
Not Applicable
Other (please specify)

Q12. At the time when you enrolled into an online course, what other support or treatment were you receiving to
manage or improve your mental health and well-being? (Select all that apply)

None
Another online program
Medication
Face-to-face therapy with mental health professional (e.g., psychiatrist, psychologist, social worker, mental health
worker)
Group therapy (including as an outpatient in a hospital setting)
Participation in an exercise group subsidised under Mental
Health Treatment Plan
Alternative medicine (e.g. naturopathy, homeopathy, acupuncture)
Not Applicable
Other (please specify)

Q13. If you didn't complete the online course, please indicate why: (Select all that apply)
I was not ready to commit to an online course at the time
I wanted to discuss it first with my health professional
I no longer felt that I needed to do the course
The cost of the course was too high
I accessed another service and/or started another treatment
I experienced technical difficulties
I didn't improve
Not Applicable
Other (please specify)

Q14. After you realised you needed support, were you able to access a mental health service or a health professional in a reasonable time?
Yes
No
Please Comment:

Q15. Were there particular qualities of the service that helped you to feel more comfortable engaging with them?
Yes
No
Please Comment:

Q16. Were there particular qualities of the health professional that helped you to feel more comfortable engaging with them?
Yes
No
Please Comment:

**Q17. Were there particular things about them that made you feel uncomfortable and not want to engage with
them?**
Yes
No
Please Comment:

Q18. Did this health professional or service help you for the length of time you felt you needed?
Yes
No
Q19. If no, did you or the health professional or service make the decision to end your support?

Myself
Service
Other
Unsure
Please Comment:
Q20. If no, did/are you intending to find alternative help for your mental health issues?
Yes
No
Q21. Do you think that disengagement (stopping) use of mental health services is an issue for a lot of people?
Yes
No
Unsure
Please explain the main reasons for your response:

Q22. Did the health professional or service give you and your family and carer sufficient notice of your impending discharge?
Yes
No
Unsure
Please explain the main reasons for your response:

Q23. Thinking about the health professional or service that you decided not to engage with (continue with) in the past, what was the primary reason you decided to stop (disengage)?
Answer choices
a. Major Contributing Reason
b. Contributing Reason
c. Not a Contributing Reason
d. Unsure
Wait times were too long
A referral was required but I didn't get one when I asked
Discharged from mental health professional/mental health service with no follow-up
The service didn't meet my needs (wrong care)
The service didn't offer me the right type of support that I needed
Cost was prohibitive/ I couldn't afford to pay for it
Limited options/choice of service providers in my area
Worker changed frequently/ no consistent worker
Told that I didn't meet/no longer met criteria of the service
Lack of plan/goals/didn't seem to be progressing/going anywhere
Didn't need the full number of appointments as I felt better quickly
Other
Please comment:

Q24. From a personal perspective, thinking about services that you decided not to engage with (continue with) in the past, what was the primary reason you decided to stop (disengage)?
Answer choices
a. Major Contributing Reason
b. Contributing Reason
c. Not a Contributing Reason
d. Unsure
Made me repeat my story too much
Didn't listen to me
Didn't include/collaborate with me

I felt I had little say or control in making decisions
Didn’t include my family/carer
My family was included and I didn’t like that
I didn’t trust them
I didn’t feel safe there
I felt judged / stigmatised by them
I was forgotten about
Decided to stop because another service was better for me
I felt better and had recovered
Decided my family or close friends supported me better
Peer support worker was best suited to my needs
Community support groups were best for me
Other
Please comment:

Q25. How did you find the communication and collaboration between health professionals and/or services?
No coordination
There was not a referral to other services
I was discharged from hospital with no referral or follow up
I was discharged from community services before I was ready
I was discharged with no other option
I felt I fell through the cracks
It wasn’t clear who I could contact when I needed to
I didn’t have a consistent person who I could contact or speak to
Each time I contacted them for help, I had to retell my story / they didn’t seem to remember my situation, needs or
preferences

Q26. If you decided not to continue but need support now or likely to in the future, which health professional or service will you try to re-engage with? Please tick all that are relevant to you
None
Psychiatrist
Psychologist
Social Worker
Public community mental health
Public mental health inpatient unit
Private psychiatric hospital
Headspace
Counsellor/therapist
GP
Peer worker
Other
Please Comment:

Q27. If you have found yourself in a crisis, would you please indicate whether any of the following contributed to the deterioration in your condition:
Couldn’t access support when needed
Didn’t have a regular health professional that I could get help from
Not connected to existing services
Regular health professional not available
Social issues
Other

Please Comment

Q28. When you found yourself in a crisis, did you seek help through an emergency department?
Yes
No
Not Applicable

Q29. If you answered yes, and you were not admitted to hospital, could you please explain what happened after you were discharged?
Went home
Went to my family or friends
No referral to mental health services
No referral to a health professional
Given a discharge letter to my GP
Given a Referral to a community mental health service
Organised consultation with community mental health team
Referral to a psychiatrist or psychologist
No follow up
Other
Please Comment

Q30. What do you think would help people stay engaged with health professionals or services or return to a health professional or service to receive support for their mental health? Please tell us your ideas

Q31. What would assist/support people to re-engage with services where they had previously disengaged from them? Please tell us your ideas

Q32. From your experience, what do people do/what happens to them after they disengage with services? Please comment:

Q33. How do you think services could best re-engage with people who have disengaged with mental health support from services? Please tell us your ideas

Q34. What services would you like to access to support your mental health and wellbeing that you can't access at the moment? Please comment

Supplementary Table S1: Frequency of Reasons for Engagement with Mental Health Services

	MAJOR CONTRIBUTING REASON (1)–	CONTRIBUTING REASON (2)–	NOT A CONTRIBUTING REASON (3)–	NOT APPLICABLE (4)–	TOTAL–
They listen to me	52.36%<br>144	32.73%<br>90	11.27%<br>31	3.64%<br>10	275
They include/collaborate with me	49.45%<br>136	31.27%<br>86	14.18%<br>39	5.09%<br>14	275
I feel safe there	50.00%<br>136	34.56%<br>94	11.03%<br>30	4.41%<br>12	272
I trust them	48.73%<br>134	36.73%<br>101	9.45%<br>26	5.09%<br>14	275
I feel I have some say or control in making decisions	48.35%<br>132	36.63%<br>100	9.89%<br>27	5.13%<br>14	273
I don’t feel judged / stigmatised by them	46.18%<br>127	35.27%<br>97	13.45%<br>37	5.09%<br>14	275
I can afford to pay for this service	44.32%<br>121	23.44%<br>64	19.41%<br>53	12.82%<br>35	273
The service meets my needs	41.30%<br>114	40.94%<br>113	13.77%<br>38	3.99%<br>11	276
They don’t make me repeat my story too much	36.40%<br>99	31.99%<br>87	25.00%<br>68	6.62%<br>18	272
I don’t have to wait too long to see someone	35.29%<br>96	34.93%<br>95	21.69%<br>59	8.09%<br>22	272
I have a consistent worker	35.69%<br>96	29.00%<br>78	13.38%<br>36	21.93%<br>59	269
Limited options/choice of service providers in my area	34.94%<br>94	21.19%<br>57	27.88%<br>75	15.99%<br>43	269
They respect my privacy if I don’t want to include my family	23.53%<br>64	26.10%<br>71	21.32%<br>58	29.04%<br>79	272
They are organised and coordinate the support services I need	20.66%<br>56	28.04%<br>76	28.04%<br>76	23.25%<br>63	271
I am able to see a worker whose gender is of my choosing	19.63%<br>53	16.67%<br>45	35.93%<br>97	27.78%<br>75	270
They seem to have a clear plan/goals	18.75%<br>51	35.29%<br>96	30.88%<br>84	15.07%<br>41	272
They include my family/carer	10.26%<br>28	10.62%<br>29	36.26%<br>99	42.86%<br>117	273

Supplementary Table S2: Frequency of Logistical Reasons for Disengagement from Mental Health Services

	MAJOR CONTRIBUTING REASON (1)-	SOMEWHAT CONTRIBUTING REASON (2)-	NOT A CONTRIBUTING REASON (3)-	UNSURE (4)-	TOTAL-
The service didn't offer me the right type of support that I needed	47.64% 101	27.83% 59	23.11% 49	1.42% 3	212
The service didn't meet my needs (wrong care)	45.75% 97	24.53% 52	26.89% 57	2.83% 6	212
Cost was prohibitive/ I couldn't afford to pay for it	42.45% 90	19.34% 41	34.43% 73	3.77% 8	212
Limited options/choice of service providers in my area	35.89% 75	25.36% 53	36.36% 76	2.39% 5	209
Lack of plan/goals/didn't seem to be progressing/going anywhere	34.78% 72	25.12% 52	35.27% 73	4.83% 10	207
Discharged from mental health professional/mental health service with no follow-up	34.13% 71	20.19% 42	42.79% 89	2.88% 6	208
Wait times were too long	27.54% 57	20.77% 43	48.31% 100	3.38% 7	207
Told that I didn't meet/no longer met criteria of the service	23.76% 48	13.37% 27	58.42% 118	4.46% 9	202
Worker changed frequently/ no consistent worker	21.95% 45	19.51% 40	52.68% 108	5.85% 12	205
A referral was required but I didn't get one when I asked	10.89% 22	13.37% 27	70.30% 142	5.45% 11	202
Didn't need the full number of appointments as I felt better quickly	4.55% 9	7.58% 15	81.31% 161	6.57% 13	198
Other	30.99% 22	5.63% 4	49.30% 35	14.08% 10	71

Supplementary Table S3: Frequency of Interpersonal Reasons for Disengagement from Mental Health Services

	MAJOR CONTRIBUTING REASON (1)	SOMEWHAT CONTRIBUTING REASON (2)	NOT A CONTRIBUTING REASON (3)	UNSURE (4)	TOTAL
Made me repeat my story too much	29.47%<br>61	27.05%<br>56	42.03%<br>87	1.45%<br>3	207
Didn't listen to me	53.55%<br>113	25.12%<br>53	20.85%<br>44	0.47%<br>1	211
Didn't include/ collaborate with me	45.93%<br>96	22.01%<br>46	30.62%<br>64	1.44%<br>3	209
I felt I had little say or control in making decisions	41.55%<br>86	26.09%<br>54	30.92%<br>64	1.45%<br>3	207
Didn't include my family/carer	11.88%<br>24	7.92%<br>16	75.25%<br>152	4.95%<br>10	202
My family was included and I didn't like that	5.50%<br>11	9.50%<br>19	80.00%<br>160	5.00%<br>10	200
I didn't trust them	38.86%<br>82	24.64%<br>52	35.07%<br>74	1.42%<br>3	211
I didn't feel safe there	40.09%<br>85	18.87%<br>40	39.15%<br>83	1.89%<br>4	212
I felt judged / stigmatised by them	47.20%<br>101	22.43%<br>48	29.44%<br>63	0.93%<br>2	214
I was forgotten about	32.21%<br>67	21.63%<br>45	44.23%<br>92	1.92%<br>4	208
Decided to stop because another service was better for me	24.51%<br>50	20.10%<br>41	51.47%<br>105	3.92%<br>8	204
I felt better and had recovered	7.80%<br>16	9.27%<br>19	79.02%<br>162	3.90%<br>8	205
Decided my family or close friends supported me better	6.90%<br>14	11.82%<br>24	76.85%<br>156	4.43%<br>9	203
Peer support worker was best suited to my needs	10.84%<br>22	11.33%<br>23	69.95%<br>142	7.88%<br>16	203
Community support groups were best for me	9.41%<br>19	15.35%<br>31	68.81%<br>139	6.44%<br>13	202
Other	19.44%<br>14	6.94%<br>5	58.33%<br>42	15.28%<br>11	72

Supplementary Table S4: Frequency of reasons for accessing GPs, ordered by major contributing reason

–	MAJOR CONTRIBUTING REASON (1) GP	CONTRIBUTING REASON (2) GP	NOT A CONTRIBUTING REASON (3) GP	NOT APPLICABLE (4) GP	TOTAL GP
They listen to me	52.88% 101	32.46% 62	13.09% 25	1.57% 3	191
They include/collaborate with me	52.36% 100	29.84% 57	15.71% 30	2.09% 4	191
I feel I have some say or control in making decisions	51.05% 97	35.79% 68	10.00% 19	3.16% 6	190
I feel safe there	50.00% 95	33.68% 64	13.16% 25	3.16% 6	190
I trust them	48.17% 92	38.22% 73	10.47% 20	3.14% 6	191
I don’t feel judged / stigmatised by them	46.60% 89	34.03% 65	15.18% 29	4.19% 8	191
I can afford to pay for this service	46.35% 89	23.96% 46	18.75% 36	10.94% 21	192
They don’t make me repeat my story too much	39.15% 74	29.63% 56	26.46% 50	4.76% 9	189
The service meets my needs	39.06% 75	42.19% 81	16.67% 32	2.08% 4	192
I don’t have to wait too long to see someone	37.57% 71	33.86% 64	21.69% 41	6.88% 13	189
I have a consistent worker	36.36% 68	28.34% 53	15.51% 29	19.79% 37	187
Limited options/choice of service providers in my area	33.87% 63	21.51% 40	29.03% 54	15.59% 29	186
They respect my privacy if I don’t want to include my family	25.40% 48	23.28% 44	20.63% 39	30.69% 58	189
They are organised and coordinate the support services I need	22.75% 43	25.40% 48	30.16% 57	21.69% 41	189
They seem to have a clear plan/goals	20.11% 38	33.86% 64	31.75% 60	14.29% 27	189
I am able to see a worker whose gender is of my choosing	19.25% 36	17.11% 32	35.29% 66	28.34% 53	187
They include my family/carer	12.11% 23	9.47% 18	34.21% 65	44.21% 84	190

Supplementary Table S5: Frequency of reasons for accessing Public Mental Health Service, ordered by major contributing reason

–	MAJOR CONTRIBUTING REASON (1) MHS	CONTRIBUTING REASON (2) MHS	NOT A CONTRIBUTING REASON (3) MHS	NOT APPLICABLE (4) MHS	TOTAL MHS
I can afford to pay for this service	57.29% 55	17.71% 17	12.50% 12	12.50% 12	96
They listen to me	40.82% 40	37.76% 37	16.33% 16	5.10% 5	98
Limited options/choice of service providers in my area	39.18% 38	27.84% 27	22.68% 22	10.31% 10	97
They include/collaborate with me	38.78% 38	35.71% 35	17.35% 17	8.16% 8	98
I feel I have some say or control in making decisions	38.78% 38	37.76% 37	14.29% 14	9.18% 9	98
I trust them	36.73% 36	38.78% 38	14.29% 14	10.20% 10	98
I feel safe there	36.73% 36	38.78% 38	16.33% 16	8.16% 8	98
I have a consistent worker	34.69% 34	32.65% 32	16.33% 16	16.33% 16	98
I don't feel judged / stigmatised by them	33.67% 33	40.82% 40	16.33% 16	9.18% 9	98
They don't make me repeat my story too much	32.99% 32	34.02% 33	23.71% 23	9.28% 9	97
The service meets my needs	29.59% 29	47.96% 47	15.31% 15	7.14% 7	98
They are organised and coordinate the support services I need	24.49% 24	29.59% 29	28.57% 28	17.35% 17	98
I don't have to wait too long to see someone	23.96% 23	36.46% 35	26.04% 25	13.54% 13	96
They respect my privacy if I don't want to include my family	21.43% 21	23.47% 23	23.47% 23	31.63% 31	98
They seem to have a clear plan/goals	17.35% 17	36.73% 36	33.67% 33	12.24% 12	98
I am able to see a worker whose gender is of my choosing	16.49% 16	23.71% 23	38.14% 37	21.65% 21	97
They include my family/carer	11.22% 11	8.16% 8	33.67% 33	46.94% 46	98
